# The use of two Comfort Young Child Formulas in the dietary management of toddlers with functional constipation: a randomized controlled trial

**DOI:** 10.1186/s12887-022-03725-0

**Published:** 2022-11-22

**Authors:** Daniel Alfonso Cisneros Sevilla, Denise Hofman, Sergio Díaz Madero, Miriam Contreras Fernández, Urszula Kudla, Eva Kontopodi, Jeske H. J. Hageman, Darelia Alelí Topete Ángel, Joshué David Covarrubias Esquer

**Affiliations:** 1 Nois de México S.A. de C.V, Guadalajara, Mexico; 2 FrieslandCampina, Stationsplein 4, Amersfoort, the Netherlands; 3 Unidad de Pediatría S.C, Mexico City, Mexico; 4 SHiEMPRE, Tonalá Jalisco, Mexico; 5 Unidad de Nutrición Infantil UNi, Guadalajara, Mexico

**Keywords:** Functional constipation, Young child formula, Hard stools, Intact protein, Dietary management

## Abstract

**Background:**

Pharmacological intervention with laxatives is the conventional treatment for functional constipation (FC). Data to support the dietary management of FC is lacking. This study compared the efficacy of two Comfort young child formulas (YCFs) with regards to the maintenance of healthy stooling parameters in toddlers with a history of constipation. It was registered in the Netherlands Trial Registry [identifier: NL7420 (NTR7653)], registration date 20/09/2018.

**Methods:**

Ninety-five healthy toddlers, aged 12 to 32 months, diagnosed with FC (Rome III criteria) were randomized to receive one of two study formulas after pharmacological treatment. For the first month of the intervention, subjects received a laxative in a decreasing maintenance dose alongside a test or control formula (maintenance phase). Subsequently, subjects only consumed formula for another month (post-maintenance phase). Stooling parameters were obtained weekly using the Bristol Stool Scale and the modified Rome III Questionnaire on Paediatric Gastrointestinal Symptoms for infants and toddlers. Differences in percentages of hard stools (primary outcome) and other stooling parameters were analysed using analysis of covariance and Chi-Square methods.

**Results:**

Both formulas resulted in similar overall percentage of hard stools during the intervention period, respectively 5.02% in the test and 2.99% in the control group (n.s.). In the test group, percentages dropped from 7.11% at the end of the maintenance phase, to 3.92% at the end of the post-maintenance phase. In contrast, the percentage of hard stools in the control group was similar at the end of the maintenance (3.18%) and post-maintenance phase (2.83%; n.s.). No difference was found in the overall stool frequency between groups. At the end of the maintenance phase, only 22% and 19% of toddlers consuming the test and control formulae, respectively, met 2 or more of the criteria for FC. At the end of the study, this percentage of subjects decreased further to 9% in the test group, which tended to be lower compared to the 21% found in the control (*p* = 0.107). No laxative use was reported in either study group during the post-maintenance phase.

**Conclusion:**

Both Comfort YCF support the maintenance of improved stooling over time in toddlers with a history of constipation. The percentage of subjects suffering from functional constipation tended to be lower after the intervention period when receiving the formula with intact protein.

## Background

Functional gastrointestinal disorders (FGIDs) are common health problems in infancy and early childhood. Even though they are mostly transient, they may be very distressing to a new parent as well as the child [1]. Gastro-oesophageal reflux (regurgitation), functional constipation (FC) and infantile colic are among the most common FGIDs, and they can occur separately or combined [2]. Both reflux and colic self-resolve by the age of 12 months, however FC often continues into toddlerhood, peaks when toilet training starts, and in extreme cases continues even to adulthood [3–5]. The prevalence of paediatric FC is difficult to determine. It is a global issue, but it varies greatly across geographical regions [6–9]. Most studies report it to range from 0.5 to 32%, with a global pooled prevalence of 9.5% [7]. FC is characterized by infrequent bowel movements, hard and/or large stools, painful defecation, and faecal incontinence, and is often accompanied by abdominal pain. FC is not caused by anatomic abnormality, inflammation, or tissue damage [10], but the exact cause is still unclear. Its pathophysiology is thought to be multifactorial. Risk factors include stress, dietary habits, physical activity, obesity, family history of FC, poor toilet training, psychological difficulties, child maltreatment, and dysbiosis of gut microbiota [11, 12]. Stooling avoidance is seen as the key factor of paediatric FC [13, 14].

FGIDs have been linked to both short- and long-term (negative) effects on health and quality of life of young children as well as their caregivers. For example, both children with FC as well as their families have lower health-related quality of life [15], while school aged- children had persistent fatigue with significant school absenteeism [16, 17]. Due to the magnitude of the problem, paediatric FC has also substantial impact on healthcare and medical costs [18, 19].

Conventional treatment for FC consists of pharmacological intervention, i.e. laxatives for stool disimpaction, in combination with non-pharmacological measures such as education and toilet training [20]. The prevailing consensus among experts is that paediatric FC should not be treated with dietary interventions [10]. This is to a great extent due to the lack of sufficient high-quality data supporting efficacy of dietary treatments [10]. However, experts also agree that proper balanced diets, with special focus on fibre and fluid intake, should be an integral part of the maintenance therapy of children that have been pharmacologically treated for FC [10, 21, 22].

The aim of this study was to assess the efficacy of two commercially available Comfort young child formulas (YCFs) with regards to the maintenance of healthy stooling parameters in toddlers with a history of FC. In addition, effects of consumption of these formulae with respect to other symptoms, defined in the Rome III criteria for the diagnosis of FC in toddlers and young children, were examined.

## Method

### Study design and population

This was a multi-centre, randomised, controlled, open-label trial, where randomization of study participants to each of the two treatment arms was based on a computer-generated sequence in blocks of 10, performed by a third-party. A total of 95 subjects were included in the study. Inclusion criteria were: I) full-term, healthy toddlers, II) with a birth weight between the 10^th^ and 90^th^ percentile, III) between 12 and 32 months of age, IV) formula-fed before and during the entire intervention period, V) with a history of hard stools, VI) with functional constipation (based on Rome III criteria), VII) parents willing to sign the written informed consent. Toddlers were not eligible for the study if they met any of the following criteria: I) diagnosed with other gastrointestinal diseases (e.g. coeliac or Hirschsprung’s disease, etc.), II) severe acquired or congenital disease, mental, metabolic of physical diseases, III) cow’s milk protein allergy (CMPA), or parents/siblings with documented CMPA, IV) perceived lactose intolerance, V) with constipation attributable to organic or anatomic causes, VI) unwillingness to stop usage of supplemented with fibres, probiotics and/or prebiotics two weeks before and during the study period, VII) considered to be a non-responder to laxatives, i.e. more than 5 times unsuccessful treatment with laxatives, VIII) medication use known or suspected to affect fat digestion, absorption and/or metabolism, IX) participation in another clinical trial, X) breastfeeding 1 month before study enrolment.

### Sample size

The sample size was calculated based on the estimated difference of 30% fewer hard stool occasions in the test group. During a two-tailed test, with an α of 5% and β of 20%, each group ideally comprised 42 subjects. Taking into account a dropout rate of 15%, 48 subjects were required per group.

### Ethics

The study protocol was approved by the ethical committee of Clínica de Enfermedades Crónicas y de Procedimientos Especiales S.C; (Michocan, Mexico) (record number: 001_2019). The information letter to parents/legal guardians and written informed consent forms were also approved by the ethical committee of Clínica de Enfermedades Crónicas y de Procedimientos Especiales S.C. (record number: 001_2019). Upon completion of the study, parents received a €50 voucher as a compensation for their time. The study was conducted in accordance with the guidelines of the Declaration of Helsinki and the International Conference on Harmonization (ICH) guidelines on Good Clinical Practice (GCP) and was registered in the Netherlands Trial Registry, [identifier: NL7420 (NTR7653)] registration date 20/09/2018.

### Study products and procedure

**Fig. 1:**
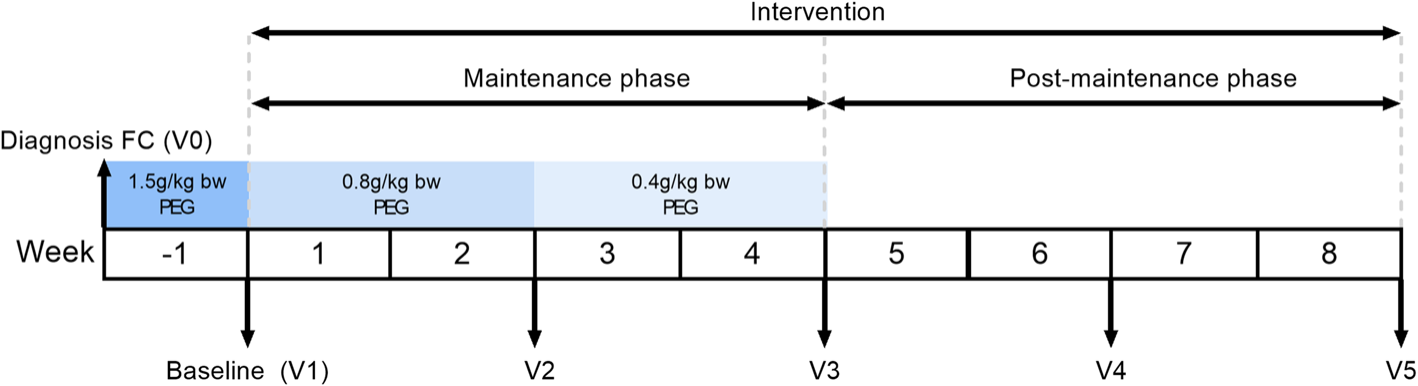
Study flow-chart (bw: body weight; PEG: polyethylene glycol; V: visit)

Pre-study treatment consisted of 1.5 g/kg bodyweight polyethylene glycol 3350 (Macrogol) (PEG). After treatment, subjects were enrolled and randomly assigned by the investigator paediatrician to receive one of two young child formulae (YCF) commercially available in Mexico in 2019, specifically designed for the dietary management of hard stools. During the first four weeks of the intervention (maintenance phase), toddlers received a decreasing maintenance dose of PEG according to standard clinical protocol, as presented in Fig. 1. Subsequently, subjects only consumed formula for another four weeks (post-maintenance phase).

The test formula (FrieslandCampina, Friso Comfort Next) contained intact protein, 20% milk fat, a fibre mixture of galacto-oligosaccharides (GOS), inulin and carob bean gum (CBG), 100% lactose and a probiotic (*B. lactis* HN019). The control formula (Abbott, Similac Comfort) contained partially hydrolysed whey (pHW), 2’-fucosyllactose (2'-FL) and reduced lactose compared to the test product. Table 1 provides an overview of the composition of both formulae, including the main functional ingredients. Parents were instructed to provide their child with three servings of formula per day. For the test product a serving size contained 180 ml with 26.4 g powder, for the control product this was 240 ml with 41.3 g powder. Three servings of the test formula provided 1.74 g of dietary fibre daily, whereas three servings of the control formula provided 1.59 g.

**Table 1:** Composition of the study formulas (per 100 g)

			**Test**	**Control**
Protein		g	21.8 (intact)	15.5 (pHW)
Fat		g	20.8 (20% MF)	23.8 (vegetable)
Carbohydrates		g	47	52.5
	Lactose	g	43.3	0.8
Total fibre		g	2.2	1.3
	GOS	g	1.2	-
	FOS			1.2
	2’-FL	g	-	0.13
	Inulin	g	0.6	-
	CBG	g	0.4	-
Probiotic			*B. Lactis* HN019	-

For the test product, one recommended serving contained 180 ml water + 26.4 g powder, for the control product, one recommend serving contained 240 ml water + 41.3 g powder

*pHW* Partially hydrolysed whey, *MF* Milk fat, *GOS* Galacto-oligosaccharides, *FOS* Fructo-oligosaccharides, *2’- FL* 2’-fucosyllactose, *CBG* Carob bean gum

At baseline (V1), subjects’ anthropometrics (body weight and length) were measured by the investigators or a research assistant and parents were asked to complete a simple food frequency questionnaire (FFQ), to assess the subjects’ habitual food intake, especially regarding dietary intake of fibres and fluids. During the intervention, parental-reported stooling parameters were obtained weekly using the Bristol Stool Scale (BSS) and modified Rome III Questionnaire on Paediatric Gastrointestinal Symptoms for infants and toddlers (QPGS-RIII, section E). Subjects, and at least one of their parents, visited the investigators every two weeks (V2, V3, and V4) to deliver all questionnaires and obtain new study products. After the eighth and final week of the intervention, anthropometrics (body weight and length) were measured by the investigator or a research assistant at the study site, and parents were asked to complete the FFQ again. Dietary fibre intake was calculated based on those results.

### Bristol Stool Scale (BSS)

The Bristol Stool Scale (BSS) is a validated, visual scale, which enables parents to provide doctors and researchers with an accurate description of characteristics of their toddler’s stools. The scale allows classification of stool form in 7 types, ranging from “separate hard lumps like nuts” (type 1) to “watery, no solid pieces” (type 7) [23, 24]. Type 1 and type 2 (“sausage-shaped, but lumpy”) stools are considered to be ‘hard stools’ and a sign of constipation. Parents were asked to complete the BSS every time their child defecated throughout the course of the study. If on any day the BSS was not completed, it was assumed that the subject did not have any bowel movements on that particular day. The BSS was used to assess overall frequency (%) of reported hard stools (type 1 and 2), overall stooling frequency, and percentage (%) of toddlers with hard stools. The frequency of hard stools was calculated as the number of hard stools divided by the number of total stools times 100.

### QPGS-RIII (section E)

The validated Rome III Questionnaire on Paediatric Gastrointestinal Symptoms (QPGS-RIII) for infants and toddlers is a parent-reported questionnaire of their child’s symptoms, useful for diagnosing Rome III diagnoses for functional gastrointestinal disorders (FGIDs), like FC (section E) [25]. In the QPGS-RIII (section E), the Rome III criteria were reworded in questions about symptoms which are understandable by parents/caregivers. The translated Rome III criteria are: defecation frequency of two or less times per week, hard or very hard stools, passing of stools during sleep, pain during defecation, large stools, stool present in rectum (as diagnosed by health care professional). For the purpose of this study, the QPGS-RIII (section E) was modified to ask parents about the past week, and parents were asked to complete the questionnaire at the end of every week during the intervention period. To be diagnosed with FC, a child should meet at least two of the Rome III criteria.

### Statistical analysis

The differences in cumulative percentage of reported hard stools (BSS type 1 and 2) (% of total stools) from the entire study period between the study groups were analysed using analysis of covariance (ANCOVA). Group was included as fixed factor, and sex, age at baseline, site, fibre intake, and fluid intake were included as covariates in the model. The same analysis was performed for the cumulative percentage of reported hard stools of the maintenance period (week 1–4) and post-maintenance period (week 5–8). Weekly stool frequency was studied with a mixed model analysis, group and visits were included as fixed factor, the interaction group*visit, and site, gender, age at baseline, fibre intake, and fluid intake were included as covariates. To study the growth during the intervention period, the change in weight and height from baseline were calculated and compared between groups with an ANCOVA. To test the differences in occurrence of the separate items of the QPGS-RIII between study groups Chi-square tests were executed. A *p*-value below 0.05 was considered to be significant. All analyses were performed using IBM SPSS Statistics version 26 (IBM Corp, Armonk, NY, USA).

## Results

### Study population

**Fig. 2:**
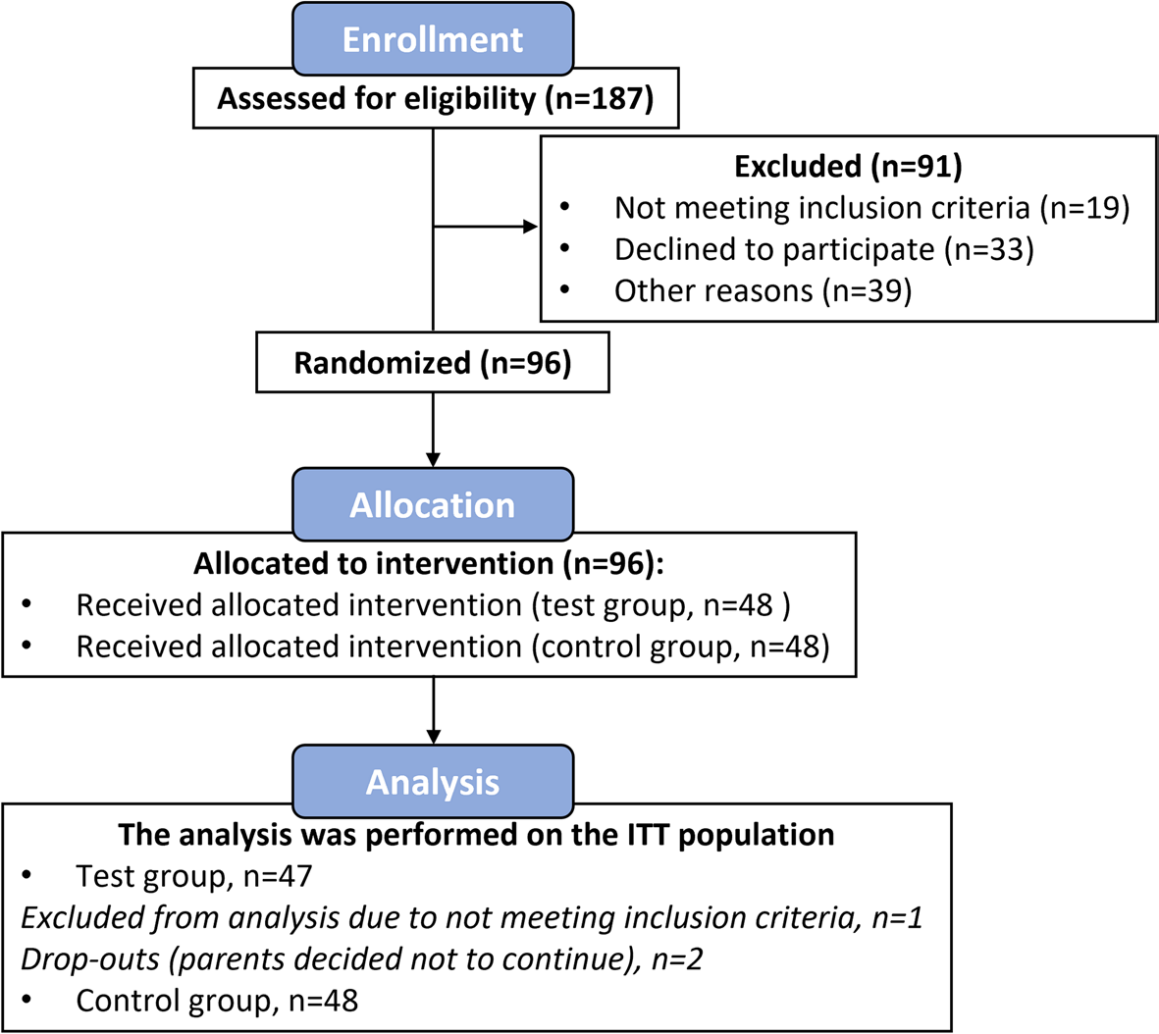
Participants flow-chart, from potential enrolment until study completion

96 subjects were enrolled in the study and randomized to a treatment, as shown in Fig. 2. One subject was excluded from analysis, as it did not meet the inclusion criteria regarding age. Two toddlers, of the test group, were lost to follow-up, as their parents decided not to continue with the study. The analysis was performed on the intention-to-treat (ITT) population, which included *n* = 47 subjects of the test group, and *n* = 48 subjects of the control group.

The demographics of both study groups are presented in Table 2. Groups were well matched with regards to gestational age, birthweight, and baseline age and anthropometrics. In addition, similar numbers of subjects in each group attended childcare or had other significant caregivers, such as grandparents, siblings, and aunts.

**Table 2:** Demographics of subjects and their caregivers, presented by study group (mean ± SD)

	**Test**	**Control**
n	47^a^	48
Gender (n)		
Male	22	27
Female	25	21
Gestational age (weeks)	39.02 ± 1.20	38.97 ± 1.08
Birthweight (grams)	3270.65 ± 407.51	3142.29 ± 376.10
Age at baseline (months)	21.12 ± 5.92	20.43 ± 6.38
Weight at baseline (kg)	11.05 ± 1.51	11.34 ± 1.90
Length at baseline (cm)	82.57 ± 6.10	82.78 ± 8.63
BMI at baseline (kg/m^2^)	16.13 ± 1.88	16.70 ± 2.74
Childcare (n)		
Yes^b^	4	5
No	43	43
Other caregivers (n)		
Yes^c^	12	13
No	35	35

^a^*n*=1 excluded from analysis based on age at baseline exceeding the inclusion criteria

^b^Childcare attendance: on average 5 days/week, range 2–7 days

^c^Other caregivers reported: grandparents, siblings, aunts

*BMI *body mass index

In addition to the study groups being well matched at baseline, assessment of the simplified FFQ as showed no significant differences in feeding practices (i.e. estimated fibre and fluid intake). Moreover, no antibiotic use was reported at any point throughout the intervention and none of the subjects were reported to use any laxatives during the post-maintenance period.

### Bristol Stool Scale (BSS)

**Fig. 3:**
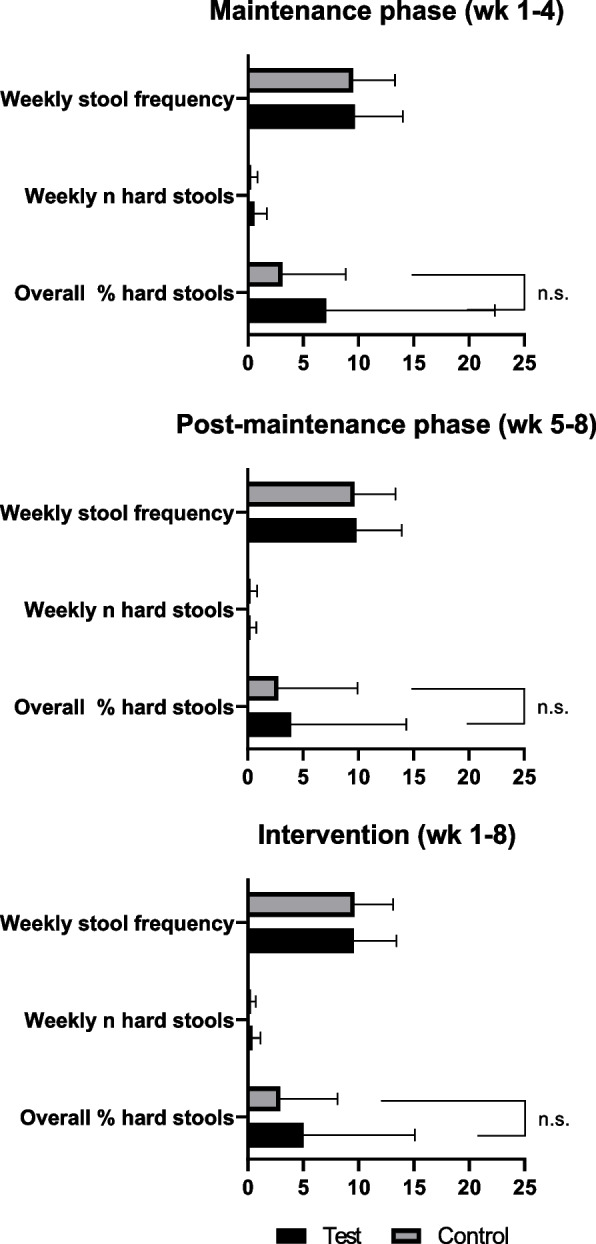
Average weekly stool frequencies, average weekly number of hard stools and overall percentage (%) of hard stools in both groups during the maintenance phase (weeks 1–4), post-maintenance phase (weeks 5–8), and entire intervention (weeks 1–8)

There were no significant between-group differences in any of the stooling parameters throughout the study. Overall, number and percentages of hard stools were low in both groups (see Fig. 3). The overall percentage of hard stools in the test group was similar to that of the control group (respectively 5.02 ± 10.06 vs 2.99 ± 5.21, *p* = 0.350). The percentage of hard stools in the control group was 3.18 ± 5.79 and 7. ± 15. in the test group during the maintenance phase (*p* = 0.154), while in post-maintenance phase it was 2.83 ± 7.22 in the control versus 3.92 ± 10.43 in the test group (*p* = 0.829). The mixed model analysis did not show any differences in stool frequency between the study groups (*p* = 0.754), nor in time (*p* = 0.434), or in the interaction between group and visit (*p* = 0.777). On average the weekly stool frequency was 9.58 ± 3.84 in the test group and 9.64 ± 3.49 in the control group. In addition, the percentage of subjects who were reported to have a hard stool on one or more occasions throughout the intervention were similar for the test (55.6%) and control (55.3%) groups. Although not significantly different, during the post-maintenance phase, the percentage of subjects reported to have hard stools was lower for the test compared to the control group (22.2% vs. 29.8%; *p* = 0.409).

### QPGS-RIII (section E)

**Fig. 4:**
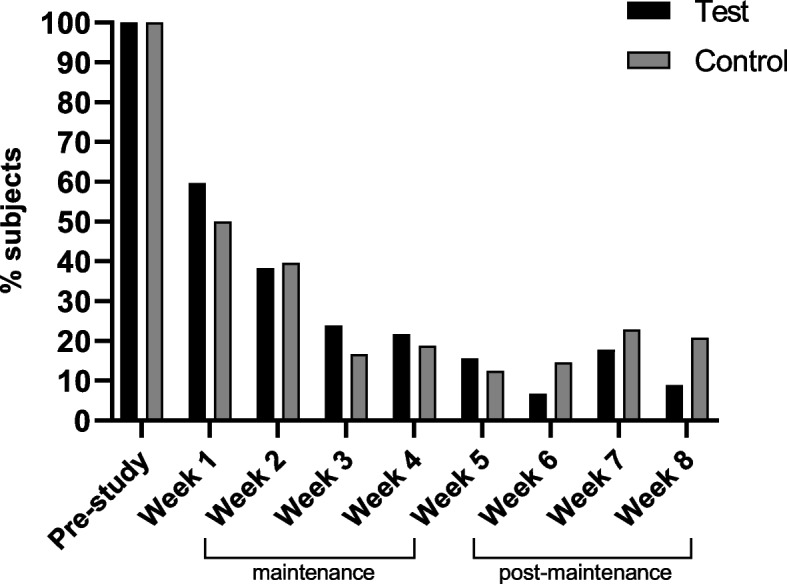
Percentage (%) of subjects with functional constipation (FC), i.e. meeting at least 2 of the Rome III criteria, during the study

Prior to the intervention, all subjects met two or more diagnostic criteria for FC (Rome III). As shown in Fig. 4, the percentage of subjects meeting at least 2 of the Rome III criteria dropped significantly after medical treatment with PEG and the first week of intervention (*p* < 0.0001). At the end of the maintenance phase (i.e. week 4), this percentage was even reduced further, 22% in the test group and 19% in the control group, but not different between study groups (*p* = 0.718). Overall, weekly percentages of subjects meeting 2 or more criteria for FC remained low throughout the post-maintenance phase, which varied between 7 and 18% in the test group, and 13 and 23% in the control group. In the test group, the percentage of toddlers meeting two or more FC criteria at the end of the study (week 8) tended to be lower compared to the control group (9% vs. 21%, *p* = 0.107).

Similar results were found when looking at individual items listed in QPGS-RIII related to FC. No significant differences between groups were observed, but all individual items significantly improved with time (*p* < 0.05), as can be seen in Table 3.

**Table 3:** Percentage (%) of subjects indicating to suffer from individual items listed in QPGS-RIII related to FC at several time-points during the study

	**Test**				**Control**			
	**Pre-study**	**Week 1**	**Week 4**	**Week 8**	**Pre-study**	**Week 1**	**Week 4**	**Week 8**
**Defecation frequency (≤ 2x/week)**	60.9^a^	8.5^b^	6.5^b^	7.0 ^b^	61.7^a^	20.8^b^	0^c^	6.3^b,c^
**Consistency (hard or very hard)**	89.1^a^	26.7^b^	2.2^c^	4.5^c^	93.6^a^	16.7^b^	2.2^b^	6.4^b^
**Faecal incontinence**^**1**^** (yes)**	37.0^a^	17.0^a,b^	4.3^b^	9.1^b^	51.1^a^	12.5^b^	14.6^b^	10.4^b^
**Painful defecation (yes)**	89.1^a^	44.7^b^	23.9^b,c^	13.6^c^	93.6^a^	36.2^b^	20.8^b,c^	12.5^c^
**Large stool (yes)**	50.0^a^	31.9^a,b^	8.7^c^	11.4^b,c^	53.2^a^	27.1^a,b^	12.5^b^	12.5^b^
**Presence of a large fecal mass in the rectum (yes)**	60.9^a^	21.3^b^	10.9^b^	9.1^b^	51.1^a^	20.8^b^	12.5^b^	4.2^b^
**Stooling avoidance**	89.1^a^	74.5^a,b^	47.8^b^	20.5^c^	78.7^a^	62.5^a^	29.2^b^	33.3^b^
Once/week	Unknown	31.9	37.0	13.6	Unknown	22.9	14.6	22.9
Several times	Unknown	36.2	8.7	4.5	Unknown	29.2	12.5	8.3
Every day	Unknown	6.4	2.2	2.3	Unknown	10.4	2.1	2.1

week 4 = end of maintenance phase; week 8 = end of post-maintenance phase

^1^passing stool whilst asleep, ^a ,b, c^ different superscript letters represent significant differences (*p* < 0.05) between weeks within study groups

### Safety parameters

Finally, no (severe) adverse events were reported throughout the study. Both groups exhibited healthy growth as indicated by mean weight-for-length (WFL) z-scores and body mass index (BMI) z-scores at the end of the intervention, as presented in Table 4.

**Table 4:** Anthropometrics of both study groups at the start (baseline) and end of study (mean ± SD)

	**Baseline**		**End of Study**	
	Test	Control	Test	Control
n	47	48	45^a^	48
Weight (kg), mean (SD)	11.05 ± 1.51	11.34 ± 1.90	11.99 ± 1.63	12.09 ± 1.90
Length (cm), mean (SD)	82.57 ± 6.09	82.78 ± 8.63	86.02 ± 5.69	86.61 ± 9.43
WFL z-score	0.31 ± 1.12	0.35 ± 1.58	0.19 ± 1.04	0.41 ± 1.46
BMI (kg/m^2^), mean (SD)	16.13 ± 1.88	16.70 ± 2.74	16.31 ± 1.84	16.49 ± 2.52
BMI-for-age z-score	0.14 ± 1.25	0.47 ± 1.58	0.16 ± 1.17	0.42 ± 1.38

*WFL* Weight-for-length, *BMI* Body mass index

^a^*n* = 2 dropouts after enrolment

## Discussion

The study reported here assessed the efficacy of two different commercially available Comfort young child formulas (YCFs) in Mexico in 2019 with regards to their applicability in the dietary management of toddlers with functional constipation (FC). Despite differences in the composition of the two study formulae (e.g. different protein fractions, fat blends, lactose and fibre content, and presence/absence of a probiotic), both study groups showed significant improvements in FC symptoms over time. As expected, total percentages of hard stools, as well as other symptoms related to FC decreased after initial pharmacological treatment with PEG. During the intervention, stooling parameters assessed in this study improved even further, and, most importantly, did not relapse throughout the post-maintenance phase. During the post-maintenance phase, none of the subjects were prescribed any laxatives. In contrast, in a study by Modin et al*.* (2018), on the use of PEG in maintenance treatment for childhood FC, the median time to the use of rescue medication in the control group, which discontinued use of PEG, was 27 days (range: 3–64 days) [26]. Therefore, lack of laxative use in our study, supports the benefits of study formulas for maintenance of good stooling parameters beyond the pharmacological treatment period. No significant differences were observed between groups. However, a tendency towards a lower percentage of subjects suffering from FC was found at the end of the study for the test group compared to the control group, respectively 9 vs 21% (*p* = 0.107). This is further substantiated by the number needed to treat (NNT) for FC, which was 8.3 in favour of the test formula at the end of the study period. NNT estimates the effectiveness of a treatment [27], and in this case it indicates that 8.3 patients need to be given treatment formula to get one more patient better, compared to control treatment.

Results showed that, during the post-maintenance phase, the overall reported percentage of hard stools (BSS) was around 3–4% in both study groups, affecting between 22.2 and 29.8% of subjects. In addition, 26.7% of subjects in the test group and 35.4% of subjects in the control group met 2 or more of the Rome III criteria for FC at some point during the last 4 weeks of the study. Previous research has shown that after two months of primarily (87%) pharmacological treatment, around 37% of young children with FC remained constipated [28]. In addition, Mill et al. stated that, even after 5 years of intensive treatment, 50% of children with FC remain symptomatic [20].

As introduced, experts believe that stooling avoidance is likely to be the most important factor for FC in toddlers and young children [13, 14]. This is due to the fact that this conditioned habitual response to painful bowel movement leads to stools becoming harder and larger and thus more painful evacuation, thus perpetuating the vicious cycle. Additionally, retained stools cause chronic distention of rectum, which will in turn lead to overflow incontinence. Amongst other individual items from the QPGS-RIII questionnaire related to FC, stooling avoidance improved significantly over time in both study groups. At diagnosis, around 80–90% of toddlers were reported to avoid stooling. At the end of the 2-month intervention, only 21–33% of all subjects were reported to exhibit stooling avoidance, of which most only once per week.

In this research we used the BSS as well as the QPGS-RIII (section E). Results showed overall low percentages of hard stools using the BSS as well as percentage of children with FC based on Rome III criteria. Whilst neither assessment tool showed significant differences between groups, there were some discrepancies in results from both measures. The number of subjects meeting two or more Rome III criteria for FC as well as the frequency of hard stools were slightly lower in the test group based on the QPGS-RIII, whilst overall percentage of hard stools was slightly lower in the control group based on the BSS. A study assessing the agreement of both measures in relation to stool consistency as well as the prevalence of FC found similar differences [29]. Authors reported fair agreement between the two methods with regards to stool consistency (κ = 0.335, *p* < 0.001). However, they did report excellent agreement between the BSS and Rome III criteria for assessing the prevalence of FC [29]. Differences in parental reports of stool consistency using these two measures might be due to the fact that the BSS provides a visual stimulus, making it easier for parents to identify the consistency of their toddler’s stool. In addition, using the BSS, consistency for every stooling occasion is recorded, whereas in the case of the QPGS-RIII, parents are presented with multiple choice answers to best describe the usual appearance of their child’s stool (e.g. “hard or very hard”, “not too hard and not too soft”). The current study had several limitations. First, the intervention period, especially the post-maintenance phase, was relatively short. Therefore, no conclusions can be drawn with regards to the long-term efficacy of Comfort YCF. Furthermore, the study was only blinded to the statistician, but not the principal investigators or parents/caregivers of the subjects, as commercial products were used. The effects observed in this study maybe specific to the tested formulas, and therefore should not be generalised to other products available on the market. Additionally, although detailed data on formula adherence were not available, the significant improvements observed over the course of the study and specifically after the laxative treatment, indicate that the consumed products were effective. In future studies, milk intake data, as well as parents satisfaction with the treatment and willingness to continue with the formula are interesting outcomes to take along. In this study a questionnaire based on Rome III criteria was used, whilst a questionnaire based on Rome IV criteria is nowadays also available. However, the criteria for FC are very similar. As also indicated by Russo et al*.*, who compared the Rome III and Rome IV criteria for the diagnosis of FC, and showed that the different criteria have a good alignment in the number of diagnoses [30]. So we believe that the results of this study are still valid in light of Rome IV, which only shows small differences with Rome III criteria. Finally, one of the three study sites, in addition to providing study formulas, educated parents and caregivers on ways to increase dietary fibre intake, whereas the other two sites did not. This did not lead to any differences in study outcomes, as neither ‘study site’ or ‘estimated fibre intake’ were found to have a significant effect in data analysis. Lack of finding any effect of potential differences in fibre intake could also be contributed to the use of a simplified FFQ, which was inadequate to properly assess dietary fibre intake.

## Conclusion

Both Comfort YCF support the maintenance of improved stooling over time in toddlers with a history of constipation. The percentage of subjects suffering from FC tended to be lower after the intervention period when receiving the formula with intact protein.

## Data Availability

The datasets used and/or analysed during the current study are available from the corresponding author on reasonable request.

## Abbreviations

2’-FL: 2’-Fucosyllactose
ANCOVA: Analysis of Covariance
BMI: Body Mass Index
BSS: Bristol Stool Scale
CBG: Carob bean gum
FC: Functional Constipation
FGID(s): Functional Gastrointestinal Disorders
FOS: Fructo-oligosaccharides
GOS: Galacto-oligosaccharides
ITT: Intention-to-treat
MF: Milk fat
NNT: Number needed to treat
PEG: Polyethylene Glycol
pHW: Partially hydrolysed whey
QPGS-RIII: Questionnaire on Paediatric Gastrointestinal Symptoms, based on Rome III criteria
RIII: Rome III
WFL: Weight-for-length
YCF(s): Young Child Formula(s)

## References

[1] Vandenplas Y, Hauser B, Salvatore S (2019). Functional Gastrointestinal Disorders in Infancy: Impact on the Health of the Infant and Family. Pediatr Gastroenterol Hepatol Nutr.

[2] Bellaiche M, Oozeer R, Gerardi-Temporel G, Faure C, Vandenplas Y (2018). Multiple functional gastrointestinal disorders are frequent in formula-fed infants and decrease their quality of life. Acta Paediatrica Int J Paediatr.

[3] Khan S, Campo J, Bridge JA, Chiappetta LC, Wald A, di Lorenzo C (2007). Long-Term Outcome of Functional Childhood Constipation. Dig Dis Sci.

[4] Afzal NA, Tighe MP, Thomson MA (2011). Constipation in children. Ital. J Pediatr.

[5] Zeevenhooven J, Koppen IJN, Benninga MA (2017). The New Rome IV Criteria for Functional Gastrointestinal Disorders in Infants and Toddlers. Pediatr Gastroenterol Hepatol Nutr.

[6] Koppen IJN, Vriesman MH, Saps M, Rajindrajith S, Shi X, van Etten-Jamaludin FS (2018). Prevalence of Functional Defecation Disorders in Children: A Systematic Review and Meta-Analysis. J Pediatr..

[7] Chogle A, Koppen IJ, Moreno JE, Ramírez Hernández CR, Saps M, Velasco-Benitez CA (2016). A Population-Based Study on the Epidemiology of Functional Gastrointestinal Disorders in Young Children. J Pediatr..

[8] AlGhamdi M, Alfetni A (2017). Prevalence and factors associated with functional constipation among children attending well baby clinic in aladel primary health care center in makkah al-mukarramah, 2016 cross sectional. Int J Adv Res (Indore).

[9] Wu TC, Chen LK, Pan WH, Tang R bin, Hwang SJ, Wu L (2011). Constipation in Taiwan elementary school students: a nationwide survey. J Chin Med Assoc..

[10] Tabbers MM, DiLorenzo C, Berger MY, Faure C, Langendam MW, Nurko S (2014). Evaluation and Treatment of Functional Constipation in Infants and Children. J Pediatr Gastroenterol Nutr.

[11] Rajindrajith S, Devanarayana NM, Perera BJC, Benninga MA (2016). Childhood constipation as an emerging public health problem. World J Gastroenterol.

[12] Avelar Rodriguez D, Popov J, Ratcliffe EM, Toro Monjaraz EM. Functional constipation and the gut microbiome in children: preclinical and clinical evidence. Front Pediatr. 2021;8:595531. 10.3389/fped.2020.595531 PMC7856458 33553067

[13] Vriesman MH, Koppen IJN, Camilleri M, Di Lorenzo C, Benninga MA (2020). Management of functional constipation in children and adults. Nat Rev Gastroenterol Hepatol.

[14] Levy EI, Lemmens R, Vandenplas Y, Devreker T (2017). Functional constipation in children: challenges and solutions. Ped Health.

[15] Rajindrajith S, Devanarayana NM, Benninga MA (2013). Quality of life and somatic symptoms in children with constipation.

[16] Wang C, Shang L, Zhang Y, Tian J, Wang B, Yang X (2013). Impact of functional constipation on health-related quality of life in preschool children and their families in Xi’an, China. PLoS ONE.

[17] Bakker RJ, van de Putte EM, Kuis W, Sinnema G (2009). Risk factors for persistent fatigue with significant school absence in children and adolescents. Pediatrics.

[18] Liem O, Harman J, Benninga M, Kelleher K, Mousa H, Di Lorenzo C (2009). Health utilization and cost impact of childhood constipation in the United States. J Pediatr.

[19] Ansari H, Ansari Z, Lim T, Hutson JM, Southwell BR (2014). Factors relating to hospitalisation and economic burden of paediatric constipation in the state of Victoria Australia, 2002-2009. J Paediatr Child Health..

[20] van Mill MJ, Koppen IJN, Benninga MA. Controversies in the Management of Functional Constipation in Children. Curr Gastroenterol Rep. 2019;21(6):23. 10.1007/s11894-019-0690-9 31025225

[21] Poddar U. Approach to Constipation in Children. Indian Pediatr. 2016;319(15):319–327. 10.1007/s13312-016-0845-9 27156546

[22] Morais MB, Vítolo MR, Aguirre AN, Fagundes-Neto U (1999). Measurement of low dietary fiber intake as a risk factor for chronic constipation in children. J Pediatr Gastroenterol Nutr.

[23] Lane MM, Czyzewski DI, Chumpitazi BP, Shulman RJ (2011). Reliability and validity of a modified Bristol Stool Form Scale for children. J Pediatr.

[24] Lewis SJ, Heaton KW (1997). Stool Form Scale as a Useful Guide to Intestinal Transit Time. Scand J Gastroenterol.

[25] van Tilburg MAL, Rouster A, Silver D, Pellegrini G, Gao J, Hyman PE (2016). Development and validation of a Rome III functional gastrointestinal disorders questionnaire for infants and toddlers. J Pediatr Gastroenterol Nutr.

[26] Modin L, Walsted AM, Dalby K, Jakobsen MS (2018). Polyethylene glycol maintenance treatment for childhood functional constipation: a randomized, placebo-controlled trial. J Pediatr Gastroenterol Nutr.

[27] Wen L, Badgett R, Cornell J (2005). Number needed to treat: a descriptor for weighing therapeutic options. Am J Health Syst Pharm.

[28] Borowitz SM, Cox DJ, Kovatchev B, Ritterband LM. Treatment of Childhood Constipation by Primary Care Physicians : Efficacy and Predictors of Outcome. 2017;115(4):873–7. 10.1542/peds.2004-0537 15805358

[29] Koppen IJN, Velasco-Benitez CA, Benninga MA, Di Lorenzo C, Saps M (2016). Using the Bristol Stool Scale and Parental Report of Stool Consistency as Part of the Rome III Criteria for Functional Constipation in Infants and Toddlers. J Pediatr.

[30] Russo M, Strisciuglio C, Scarpato E, Bruzzese D, Casertano M, Staiano A (2019). Functional Chronic Constipation: Rome III Criteria Versus Rome IV Criteria. J Neurogastroenterol Motil..

